# How do protected landscapes associated with high biodiversity and population levels change?

**DOI:** 10.1371/journal.pone.0180537

**Published:** 2017-07-03

**Authors:** Pablo Cuenca, Cristian Echeverria

**Affiliations:** 1 Universidad Regional Amazónica IKIAM, Tena, Ecuador; 2 Laboratorio de Ecología de Paisaje, Facultad de Ciencias Forestales, Universidad de Concepción, Concepción, Chile; Universidad de Chile, CHILE

## Abstract

Most protected areas (PA) try to limit logging of forests by means of restrictions on access and use, especially in areas where local communities coexist with the forests and depend on resources derived from PAs. In such contexts, achieving full or effective protection of the forests is almost impossible. This fact has led to researching beyond PAs boundaries in order to examine large surrounding landscapes with multiple forms of properties and restriction on forests use. The present study assessed the change in forest cover and fragmentation between 1990 and 2014, in addition to the drivers that explain such changes in a landscape with the presence of PAs and high-density population belonging to the Chocó-Darien biodiversity hotspot. Results indicated differences in the extent and spatial patterns of change in forest cover of PAs and their surrounding landscapes. Two PAs exhibited a tendency to increase fragmentation and lose their forests in comparison with the stable protection of the forests in other PAs during this period. However, the greatest change in forest cover and fragmentation was observed in the surrounding landscapes, where the best connection to markets and transport networks were the dominating deforestation drivers. Our findings corroborated that the PAs were a shield against the deforestation of the tropical Andean forest, especially in landscapes with high-density population. However, the fragmentation of the forest cannot be avoided around the PAs limits. It is expected that, if this tendency continues in the future, the biodiversity in the Chocó-Darien hotspot will be seriously affected.

## Introduction

Protected areas (PA) are recognized as a cornerstone to maintain and ensure the biological conservation of the planet [1]. It is estimated that approximately 209,000 PAs were established in 193 countries and territories in 2014, protecting 17% of the global terrestrial surface [2]. The goal of most PAs is to limit the logging of forests by means of restrictions on access and use, especially in areas where local communities co-exist with forests and depend on resources derived from the Pas [3, 4]. In such contexts, achieving full or effective protection of the forests is almost impossible [5–7].

Rapid losses in forest cover and fragmentation around PAs have been reported in the tropics [3, 8], especially due to human pressure in landscapes surrounding the PAs [9]. This fact has resulted in the isolation and reduction of forest habitats and the impact on the ecological processes in PAs [10]. Even when the forest cover is not affected, changes in the landscapes that surround the PAs can significantly impact the flow of species and energy and cause disturbances due to greater exposure to human impact [3, 11, 12].

The temporal analysis of fragmentation and cover change in forests using satellite images has become a valuable technique for assessing the degree of threat posed to protected and non-protected forest ecosystems [13–16]. There are various studies on deforestation patterns based on images that have been obtained in tropical forests [17–20] and in temperate forests [16, 21, 22]. On the other hand, there are very few studies on landscapes with tropical Andean forests and the presence of PAs [17, 23, 24], even though these ecosystems are recognized as some of the most megadiverse ecosystems worldwide. Myers, Mittermeier [25] and Pimm, Jenkins [26] stated that these forests constitute a "hotspot" of biological diversity which are disappearing as a result of the rapid process of change in land use to meet the demand for wood and non-timber forest products, among others.

There is a pressing need to identify the causes of forest fragmentation and deforestation to understand how these processes affect the spatial configuration of the landscape over time [27–29]. There is the consensus that the simple description of the types of forest cover are inadequate for the planning of forest resources, because there is no information about the change patterns of soil use that can deeply affect the ecological processes of interest [30, 31]. Therefore, it is necessary to study the factors responsible for deforestation to have a more comprehensive understanding of protected landscapes. This goal involves a comprehensive analysis of the processes and not just patterns beyond the limits of the PAs, in order to examine large surrounding landscapes with multiple forms of properties and restrictions on forest use [32].

The tropical Andean forest of Ecuador offers a particularly instructive example of the PAs-individuals interactions in landscapes with high biodiversity (hotspots) [25, 26, 33, 34] and marked tension between conservation and development, which have been increasing in recent years [4, 35–37]. Examples are Mache-Chindul Reserve (MCR), Cayapas-Mataje Mangroves Reserve (CMMR), and Cotacachi-Cayapas Reserve (CCR). The human communities settled within these reserves and the surrounding landscapes coexist with high biodiversity levels and, at the same time, access to forest resources, that are part of their cultural, social, and institutional interactions with nature, is restricted [14, 38]. Despite the biological importance of these ecosystems [26, 39], little is known about the patterns of deforestation and fragmentation of the tropical Andean forest cover, and which social and environmental factors explain such changes.

In the present study, we assessed the rates and patterns of loss and fragmentation of the tropical Andean forest in the MCR, CMMR, CCR, and the surrounding landscapes. Additionally, we identified the social and environmental driving forces that determine the processes of the landscape related to the change in the protected landscapes.

## Materials and methods

### Area of study

The forests of northwestern Ecuador have been catalogued as one of the areas with greater risk of biological extinction resulting from deforestation and anthropogenic activities [40–42]. Of the approximately 80,000 km^2^ of native forest, which originally covered the Ecuadorian northwest, just 6% was preserved at the beginning of the 1990's [43].

**Fig 1:**
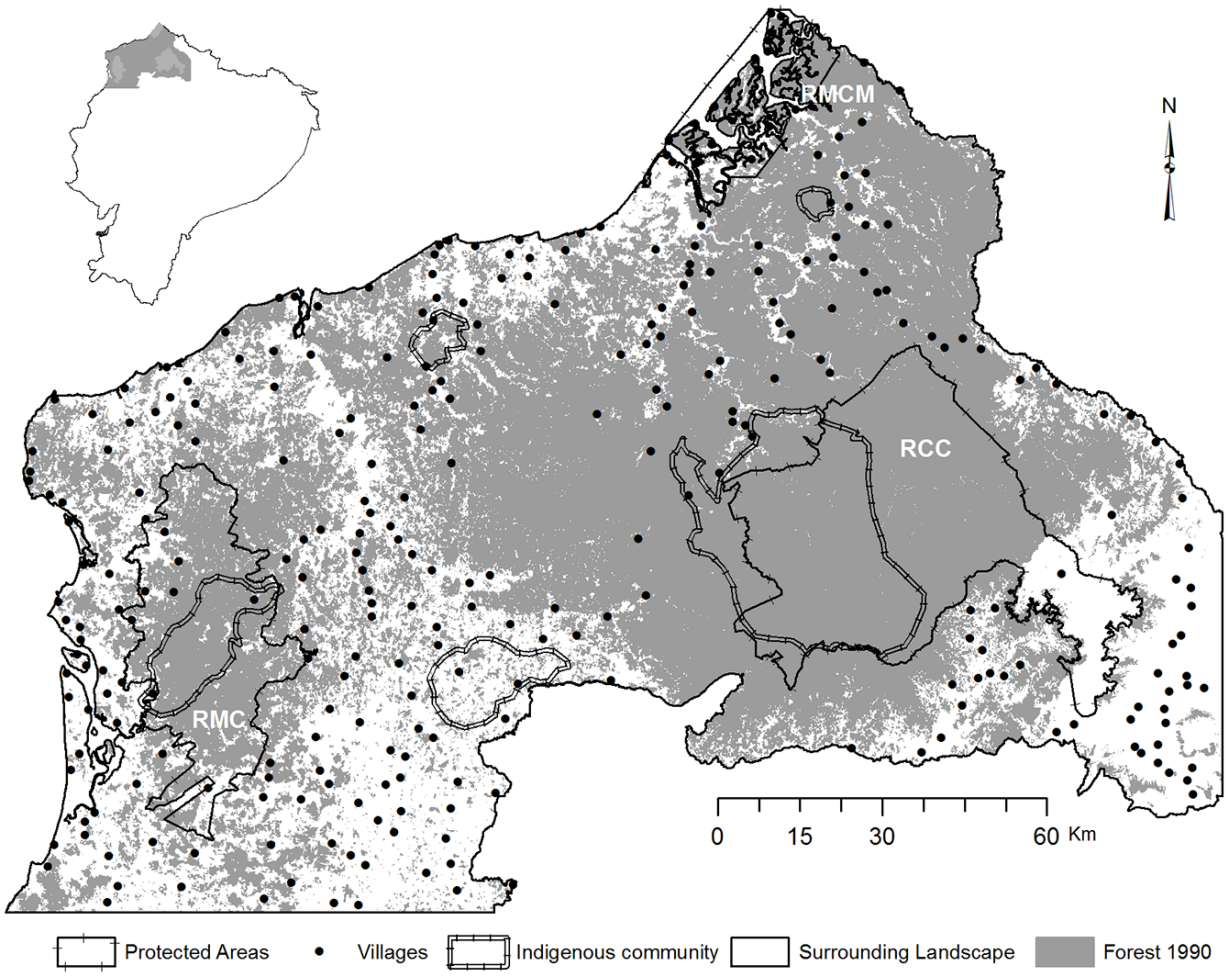
Location of the study area in the protected landscape.

The study area is located in northwestern Ecuador and is part of the Choco-Darién hotspot with biodiversity at global level. This hotspot includes the PAs MCR, CMMR, and CCR, and is characterized by high level of biological diversity, endemism, and destruction of natural habitats [25, 26] (Fig 1).

The MCR is located in the vegetation formations of the upper montane evergreen forest and lowland evergreen forest, with gentle to steep slopes. The CMMR covers the vegetation formations of the lowland evergreen forest, flooded lowland evergreen forest, and mangroves. The CCR protects seven vegetation formations [43, 44].

According to the 2010 population census, of the inhabitants registered in the counties that constitute the MCR, 32% inhabited the reserve (6,466 inhabitants) and 18,159 ha belonged to Los Chachi community, i.e., 15.23% of the MCR territory [43]. In the CMMR, 29% of the terrestrial surface belonged to private lands and 8.4% corresponded to community lands (Afro-Ecuadorians and Chachi communities). In 2005, it was estimated that approximately 24,000 Afro-Ecuadorians, 3,500 Chachi indigenous individuals lived around and within the reserve, in addition to the peoples Kichwa, Awá, and Épera, with a number of inhabitants not officially quantified [45–47].

### Analysis of the change in forest cover

The baseline information used consisted of satellite images (Landsat-5 TM) obtained in 1990, 2000, 2008, and 2014, which were classified by the Ecuadorian Ministry of the Environment through the Deforestation Baseline Project and the Socio Bosque Program [48].

A single image was created with the forest cover. It included the changes in the trajectories or sequences of forest cover types observed in the observation periods. The changes in the trajectories of forest cover were grouped into four categories. Pixels that changed from non-forest to open forest and from non-forest to closed forest were treated as regeneration. Pixels that changed from closed forest to non-forest and from open forest to non-forest were considered deforestation. In contrast, pixels that had maintained open forest or closed forest in the two periods of analysis were considered persistent forest. The pixels related to non-forest in the two periods were not included in the study.

Following the approaches often used in other studies, such as those proposed by Ewers and Rodrigues [49], Gaveau, Epting [15], and Nagendra, Paul [3], we defined the surrounding landscapes beyond the limits of the PAs taking into account the need to compare non-protected wide areas in surrounding landscapes with PAs. The extension of the surrounding landscapes was the administrative-political division where the PAs were located, because this division covered a broad landscape with similar characteristics of land use, land ownership, population, and natural resource management.

### Loss of native forest

The maps of forest cover change and the quantification of native forest loss in the PAs and the surrounding landscapes were carried out using ArcGIS spatial analysis (version 10.2.2). The category native forest was used to perform the analysis of deforestation. The formula used to determine the annual deforestation rate was that proposed by [21] and [22]:

$$P=\text{[}{\left(\frac{A_{2}}{A_{1}}\right)}^{1/\left(t_{2}-\;t_{1}\right)}-1\text{]}*100$$

Where *A*_1_ and *A*_2_ are the forest cover in time *t*_1_ and *t*_2_, respectively; and *P* is the percentage of loss per year.

### Deforestation drivers

A logistic regression analysis was used to analyze the deforestation driving forces. Image maps of forest cover obtained in the periods 1990–2000, 2000–2008, and 2008–2014 were superimposed in a geographical information system (GIS), and each pixel of the image was rated both as persistent forest and deforestation. A set of 4921 points or pixels equidistant to 1,500 m were randomly inserted for the PAs and the surrounding landscapes, respectively. The degree of spatial autocorrelation of the sampling points were assessed using Moran’s index, which provided a value of 0.36, where 0 indicated null spatial dependence and 1 indicated high degree of spatial autocorrelation [50].

The models were adjusted using the generalized linear model [51, 52] with a binary variable (1 = deforested pixels, 0 = forested pixels) linked to a logit function and a linear combination of the following explanatory variables: slope (°); elevation (m); distance to national roads (km); distance to local roads (km); distance to permanent rivers (km); distance to secondary rivers (km); distance to villages (km); temperature (°C); and average rainfall (mm).

In the model, we assessed whether the exploratory variables affected the probability of deforestation using a GLM fit, and tested the statistical significance of each variable using the z value test. Subsequently, all the variables with 95% significance (*p* <0.05) were subjected to multivariate analysis in order to test whether the variables with significant values could be reduced due to the covariance between them. The drop1 function was used to test whether the change in the variance associated with the abandonment of the terms of the model was significant (X^2^ test). We used the drop1 function to obtain a parsimonious model in which all the terms were significant (*p* <0.05).

### Landscape fragmentation

We assessed the spatial patterns of forest cover using the Fragstats 4.2 software [53], because it provides a powerful and comprehensive set of descriptors of spatial patterns. The following class indexes were considered for such purpose: (i) size of patch (area in hectares); (ii) index of the closest neighbor (the distance to the closest edge in meters between a patch and its closest neighbor of the same category); (iii) index of mean form, which refers to mean complexity for a category; and (iv) patch density, which corresponds to the number of patches by hectare. For more details of the metrics see [53]. These indexes were compared to assess whether they differed between the cover of native forests within PAs and the surrounding landscapes.

## Results

### Forest cover change

**Fig 2:**
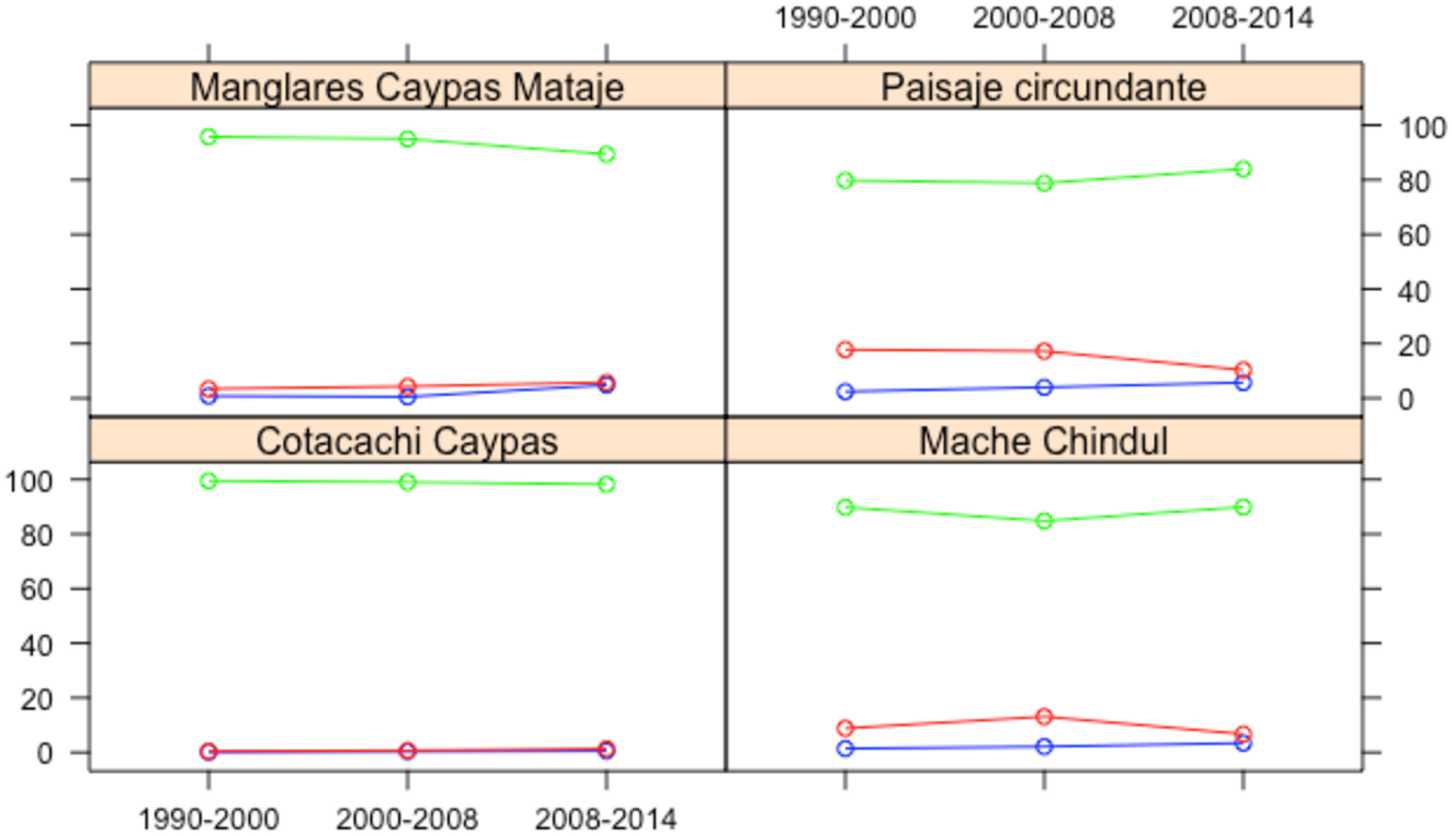
Percentage of forest cover change between 1990–2000, 2000–2008, and 2008–2014 in different protected areas within the landscape. *Note*. green = persistent forest; blue = regeneration; red = deforestation.

We observed a significant loss of native forests in the MCR and CMMR. It was from 8.8 to 13.1% in the period 1990–2000 and 3.4 to 4.6% in the period 2000–2008. In the MCR, the deforestation tended to decrease slightly (6.7%) in the period 2008–2014. On the other hand, it increased to 5.8% in the CMMR. In these two reserves, the persistent forest showed no regeneration during the two first periods, with a light recovery in the third period. In the CCR, the deforestation rate was low in the three periods of analysis (less than 1%), except in the last period, in which deforestation increased slightly. In this PA, the persistent forest and the regeneration were constant (Fig 2). An opposite tendency was observed in the surrounding landscapes in the period 1990–2000. There was a deforestation of 17.8%, which was a tendency maintained almost equal in the following period, though not in the period 2008–2014, when the deforestation decreased to 10.3%. The persistent forest and regeneration increased only in the last period of analysis.

**Fig 3:**
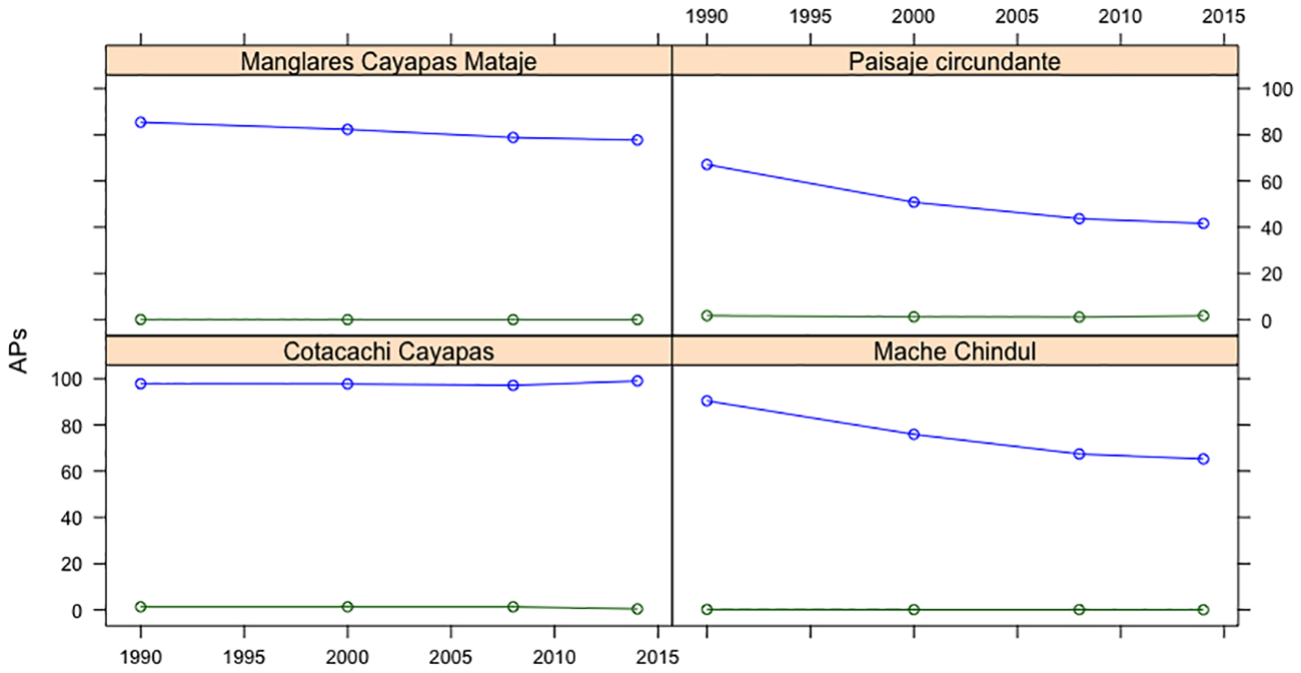
Percentage of area occupied by type of forest cover in 1990, 2000, 2008, and 2014. *Note*. blue = closed forest; green = open forest.

There was a predominance of closed forest followed by open forest from 1990 to 2000 in the three reserves and the surrounding landscapes (Fig 3). Specifically, in the MCR, forest cover was 90.4% of the total PA in 1990, substantially decreasing to 76, 67, and 65% in 2000, 2008, and 2014, respectively. A similar tendency was observed in the CMMR, where the closed forest represented 85% of the total PA in 1990. There was a slight decrease in the following years, i.e., 82 to 79% from 2000 to 2008 and to 78% in 2014. An opposite tendency was reported in the CCR, where the closed forest represented 98% of the PA in 1990, with a slight reduction to 97% in 2000, followed by a low reduction to 96% in 2008, and regeneration of 99% in 2014. The surrounding landscape showed an expected tendency. The closed forest occupied approximately three-quarters of the PA in 1990, with a significant decline from 51% in 2000 to 42% in 2008. In the last year of analysis, the closed forest exhibited a slight reduction to 41% of the total area of study.

The deforestation rate in the MCR and CMMR in the three periods of analysis increased, except for the period 2008–2014, in which the rate only decreased in the MCR (Table 1). The deforestation for in the CCR was very low from 1990 to 2008; however it exhibited a slight decrease in the period 2008–2014, causing a loss of 2,539 ha in the native forest. On the other hand, the deforestation rate in the surrounding landscapes was high and showed a tendency to increase in the three periods of study (Table 1).

**Table 1:** Change in the surface of the native forest in 1990, 2000, 2008, and 2014.

Categories	Mache-Chindul Reserve	Cayapas-Mataje Mangroves Reserve	Cotacachi-Cayapas Reserve	Surrounding landscapes
Native forest cover in 1990 (ha)	98,384	28,957.41	202,694	1,039,705
Native forest cover in 2000 (ha)	90,980	28,001.88	202,821	871,624
Native forest cover in 2008 (ha)	80,831	27,017	202,501	752,515
Native forest cover in 2014 (ha)	78,167	26,074	202,618	654,118
Change rate in forest cover 1990–2000 (% per year)	0.78	0.33	-0.006	1.75
Change rate in forest cover 2000–2008 (% per year)	1.47	0.45	0.02	1.82
Change rate in forest cover 2008–2014 (% per year)	0.56	0.59	-0.1	2.3

### Change in spatial patterns

**Fig 4:**
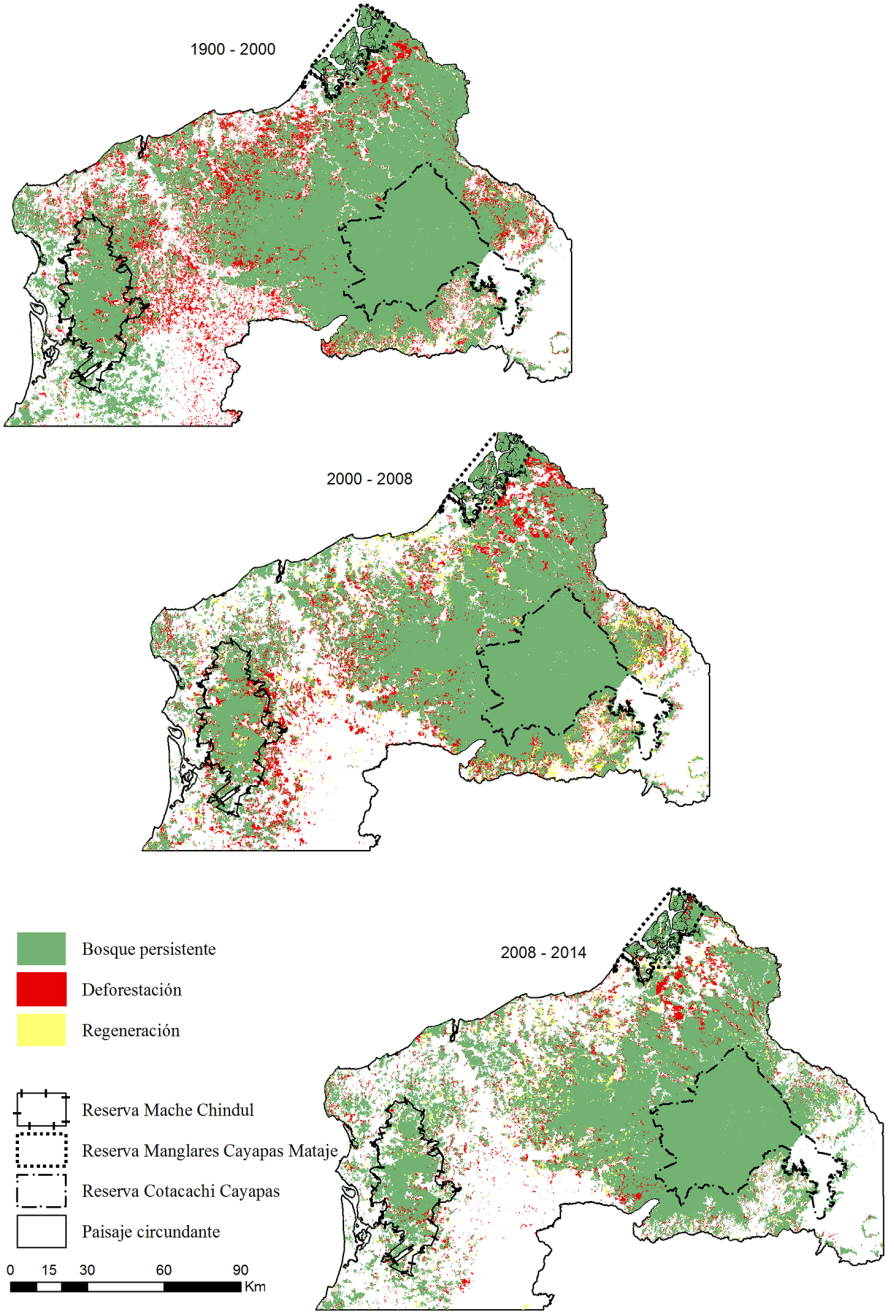
Spatial-temporal changes in forest cover of the protected landscape.

Even though there was regeneration in the landscapes, great part of it seems to have occurred in areas towards the centre of the surrounding landscapes, where there were less villages (Fig 4). Lower regeneration was observed to the southeast of the CCR due to the abrupt topography that hinders logging. We also observed regeneration of forests in the limits of the MCR, particularly in the north between 2008 and 2014, indicating the old areas of forest exploitation that have been discontinued and were under greater regulations. The long patches of persistent forests that remained to the north of the CCR indicated in some way the impact of the forest protection, in addition to the steep topography especially to the northeast of this AP that decreased the large-scale wood extraction. Although it was observed that most of the deforestation occurred in non-protected areas of the surrounding landscapes, there was also forest loss in the edge of the PAs where access was easier. In the MCR, deforestation mainly occurred in the eastern and central regions, where the protected forest was surrounded by densely populated communities and villages connected to the road network. On the other hand, deforestation was more pronounced to the southern and eastern regions of the CCR and CMMR, respectively (Fig 4).

**Fig 5:**
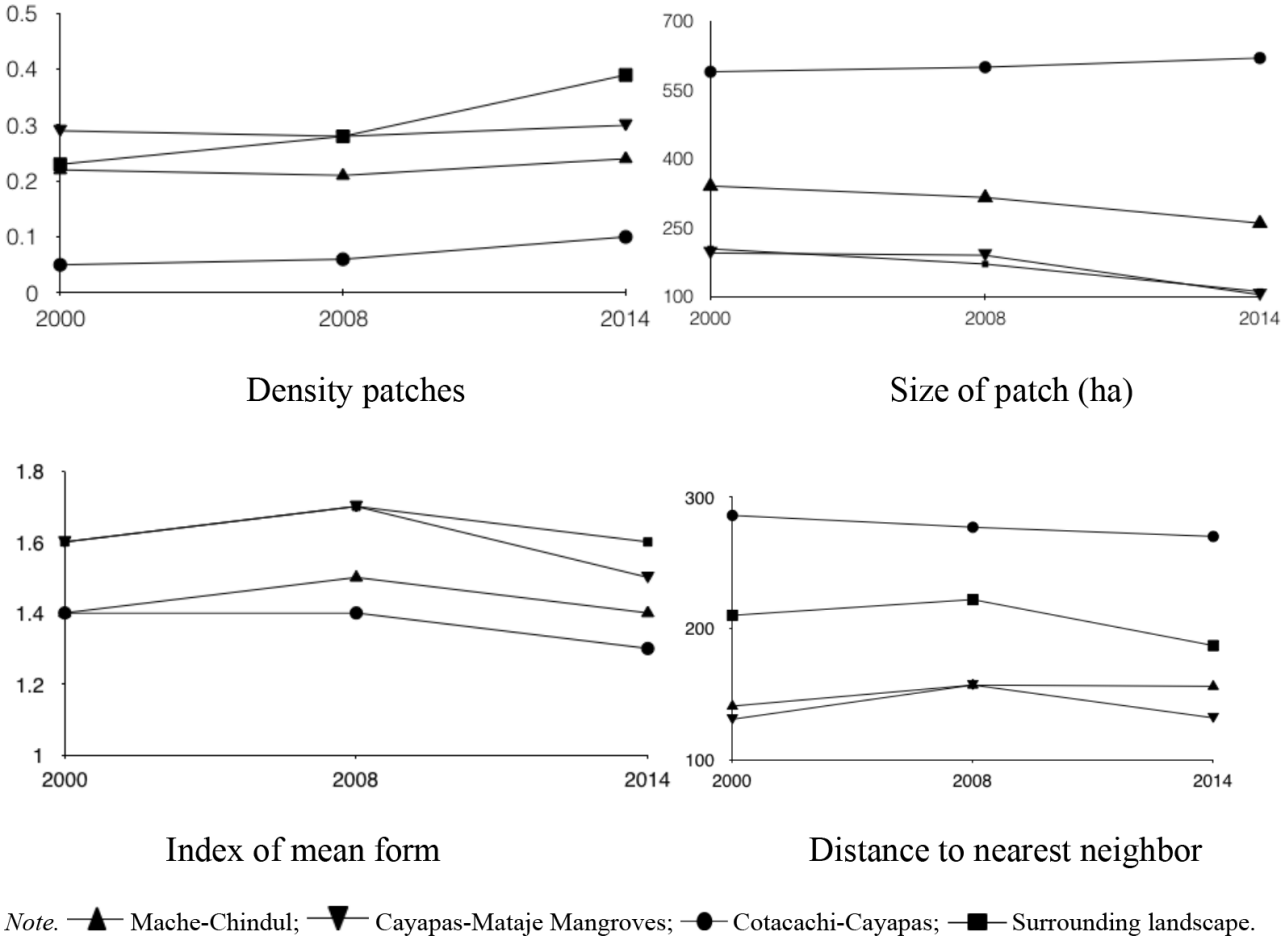
Metrics per class in the persistent forest.

The fragmentation of the persistent forest is shown in Fig 5. The persistent forest was apparently more fragmented in the surrounding landscape, with a small average patch area. low average index values, high-density patches, and a distant location (average distance to nearest neighbor). On the other hand, between the APs, the less to more fragmented forest cover was in the CCR, followed by the CMMR and MCR, respectively.

### Deforestation drivers

Slopes, distance to secondary roads, distance to secondary rivers, and distance to villages were significantly and negatively related in the period 1990–2000 (Table 2). In the same period of study, the distance to PAs were positively related to deforested areas. For the following period (2000–2008), the logistic regression analysis showed that slopes, distance to secondary roads, distance to secondary rivers, and distance to villages were significantly associated with deforestation areas. On the other hand, temperature was positively related to the probability of deforestation. In the period 2008–2014, slope, distance to secondary roads, distance to main roads, distance to secondary rivers, and distance to villages were significant and negatively related to deforested areas (Table 2).

**Table 2:** Exploratory variables used to estimate the probability of deforestation in the landscape of study.

Variables	Coefficients	Standard Error	z value	*p*-value
*Period 1*: *1990–2000*				
Intercept	1.720e+00	1.570e-01	8.298	**
Distance to secondary rivers	-2.586e-04	3.147e-05	-8.217	**
Slope	-2.271e-02	3.998e-03	-5.680	***
Distance to villages	-1.293e-04	1.639e-05	-7.892	***
Temperature	3.017e-02	2.137e-02	1.412	NS
Distance to main roads	-2.445e-05	4.105e-06	0.956	NS
Distance to permanent rivers	7.492e-06	4.751e-06	1.577	NS
Rainfall	-1.373e-04	4.591e-05	-2.991	NS
Altitude	-2.352e-04	1.694e-04	-1.388	NS
Distance to protected areas	1.576e-05	3.290e-06	4.789	**
Distance to secondary roads	-4.384e-05	5.389e-06	-8.136	***
*Period 2*: *2000–2008*				
Intercept	-1.510e+00	8.235e-01	-1.833	NS
Distance to secondary roads	-6.458e-05	4.699e-06	-13.744	***
Slope	-1.180e-02	3.660e-03	-3.225	**
Distance to villages	-9.331e-05	1.389e-05	-6.718	***
Temperature	5.549e-02	1.130e-02	2.911	NS
Distance to main roads	1.385e-06	3.888e-06	0.356	NS
Distance to permanent rivers	8.900e-06	8.199e-06	1.085	NS
Rainfall	1.109e-04	4.364e-05	-2.542	NS
Altitude	5.808e-05	1.956e-04	0.297	NS
Distance to protected areas	1.061e-05	3.473e-06	-3.055	NS
Distance to secondary rivers	-1.679e-04	1.996e-05	-8.413	***
*Period 3*: *2008–2014*				
Intercept	1.933e-01	1.082e-01	1.788	NS
Distance to secondary roads	-4.660e-05	5.465e-06	-8.527	***
Slope	-2.854e-02	4.355e-03	-6.555	**
Distance to villages	-8.150e-05	1.633e-05	-4.990	***
Temperature	2.007e-02	2.309e-02	0.870	NS
Distance to main roads	-2.154e-05	4.463e-06	-4.827	**
Distance to permanent rivers	5.003e-06	9.933e-06	0.504	NS
Rainfall	7.186e-05	4.641e-05	1.549	NS
Altitude	-3.532e-04	1.831e-04	-1.930	NS
Distance to protected areas	-6.044e-06	4.079e-06	-1.482	NS
Distance to secondary rivers	-1.472e-04	2.234e-05	-6.588	***
*N* = 4921 points; n = 1065 deforested points; n = 3856 forest points; df = 1				

Note:

**, *** indicates significance at 5% & 1%, respectively.

NS indicate not significant

The non-significant variables in the protected landscapes in the period 1990–2000 were: temperature; rainfall; distance to main roads; distance to permanent rivers; and altitude. In the following period, the variables were: distance to main roads; distance to permanent rivers; altitude; rainfall; and distance to PAs. In the period 2008–2014, the non-significant variables were: distance to permanent rivers; altitude; distance to PAs; and temperature.

## Discussion

The present study assessed whether the PAs have experienced different patterns of change in forest cover and fragmentation in comparison with non-protected surrounding landscapes. These types of studies are complementary to determine the effect of PAs on deforestation, since they are analyzed in a context of large changes and landscapes within which the PAs are embedded [3, 54, 55].

### Loss of native forest

The results of the present study revealed the accelerated forest loss in PAs and in their surrounding landscapes in the last 24 years, compared with the findings of others studies conducted in the tropics [3, 13, 15, 19]. The annual rate of deforestation in the surrounding landscapes from 1990 to 2000 was almost two times higher than the rate reported by [56] and [57]. In the second period (2000–2008), the rate of deforestation increased even more, surpassing the rate reported in the northern Ecuadorian Amazon by [19]. In the third period (2000–2014), the rate of deforestation maintained its tendency and increased almost 5%, which was the highest rate of deforestation reported in the Ecuador when compared with the findings of other studies [20, 58–60].

In the surrounding area, in addition to the agricultural activity, especially oil palm cultivation in land of gentle slope, there are oil refineries that have contributed to the construction of new roads, thus accelerating the process of colonization of new lowland areas [43]. These facts have probably been the leading causes of forest loss in the protected landscapes.

Similar processes occurred in the northeast of Ecuador [20]. The Agrarian Reform of Ecuador in 1964 promoted the colonization and logging of areas, especially to demonstrate possession of the land by the settlers [60–62]. This fact has contributed to the increase in the population of the surrounding landscapes and, consequently, caused higher pressure to the forest, since many settlements depended on the forest for their subsistence [5, 63].

In the MCR, the rate of forest loss was high, even matching the rate of deforestation in the surrounding landscape in the period 1990–2000. In the MCR and CMMR, the rate of deforestation maintained the same tendency in the second period (2000–2008) and increased almost the double in the last period (2008–2014). The CCR exhibited an opposite tendency, i.e., a decrease in the rate of deforestation. In the first two periods, the increase in the rate of deforestation would have resulted from a higher density of human settlements, increasing accessibility, and connectivity of roads and secondary rivers surrounding the area and in the MCR. On the other hand, in the last period and in this protected area, the decrease in the rate of deforestation could have been related to a positive economic situation due to the oil industry, which allowed increasing the budget in all the National System of Protected Areas of Ecuador in 2008 and 2014 [4].

In the CMMR, the tendency to increase the rate of deforestation was probably due to strong pressure exerted by the human settlements in the surrounding area [3, 64]. In the CCR, low deforestation was probably due to the fact that agricultural and livestock activities require gently sloping terrains [65], a condition that was not found in the CCR. In addition, the low pressure exerted by the few human settlements and high elevation favor the persistence of the forest.

Even though in Ecuador the reduction of forest loss is supported by its new Constitution, the present study revealed that the rate of deforestation in the surrounding landscapes exhibited a tendency explained by extraction and commercialization of wood in illegal markets, especially in populations settled in the province of Esmeraldas [60].

However, it was also noted that there was a tendency to reduce the rate of deforestation in the PAs due in part to the effort made by the Department of Forest Control of the Ministry of the Environment of Ecuador. Despite this effort, significant amounts of illegal timber are confiscated from individual poachers and settlers [66]. It is clear that the pressure exerted in the PAs by the communities was still significant and the authorities had difficulties in fully protecting the forests.

### Patterns of fragmentation

The present study provides evidence that the fragmentation continued in forest habitats due to increasing patches density and the isolation of the tropical Andean forest. The constant dependence of the settlers on the forest, especially in the settlements along secondary rivers and roads, was causing small and irregular fragments. In addition, the opening of the paved road between Pedernales and Muisne―locations within the landscape under study―would have probably caused significant increase in fragmentation of the protected and non-protected forests, since it has been reported that the destruction and fragmentation of the remaining native forest have accelerated in it last years due to the opening of the roads. It is worth mentioning that similar patterns have been reported in the tropics [10, 13, 67].

Marquette [68] reported that there was a combination of livestock and small-scale agricultural activities in the northeast of Ecuador. In this tropical ecosystem, approximately 80% of small farmers cut down small areas of forest [19, 68]. The northwestern forest of Ecuador studied was one of the last remnants of tropical Andean ecosystems distributed in an average patch of 1.39 km^2^ in the surrounding landscape during the three periods studied. This fact means that fragmentation was high and a key process around the PAs.

Despite the fact that deforestation in the PAs showed a tendency to decrease over time, it was not the case of fragmentation patterns, which have been increasing over the years. The establishment of the PAs in this landscape has been a key to reduce forest loss [24]. However, the high rates of continuing deforestation and fragmentation in the surrounding landscape puts at risk the maintenance of the persistent forest and leads to the isolation of patches in the forest. In such context, achieving complete or effective protection of the forest is increasingly difficult [5–7] and, therefore, there is an expected reduction in the ecological integrity and the ability to preserve this hotspot of biological diversity [9, 39].

### Deforestation drivers

The areas with gentle slopes had a greater probability of deforestation due to the expansion of lands for palm oil cultivation and livestock [69]. These results are in line with those reported by Laurance, Albernaz [13] and Wilson, Newton [70], who stated that the slopes were a highly significant variant to explain the probability of deforestation, both in temperate and tropical ecosystems. The distance to secondary roads and rivers were highly significant to explain forest loss, since it is assumed that areas closer to roads are more accessible to be deforested. In the area studied, the rivers were a very important source of communication and transport of legal or illegal wood. In the three periods of analysis, the distance to populated areas were highly significant, corroborating the strong anthropic impact that occurred in this landscape. In all the periods studied, temperature and rainfall were not significant, which is consistent with the findings reported by Geldmann, Barnes [65] and Green, Larrosa [71].

### Implications for conservation

There is a clear need of involving the local inhabitants with the efforts for conservation in order to ensure the persistence of the tropical Andean forest in this region. As shown in the present study, there was a decrease in forest loss in the CCR and CMMR, probably attributable to factors such as high population density in this protected landscape, high level of social and ethnic heterogeneity―that has triggered the increase in migration in recent years―and the lack of new benefits provided by development partnerships in the area [60, 72]. In this context, development initiatives in the region provided an impulse for the local economy and also posed a significant threat in the extension and connectivity of forest cover in this protected landscape [19].

There has been a considerable interest in understanding the impact of the PAs on the change in forest cover, especially in the tropics due to the implications of these ecosystems for the mitigation of climate change [73, 74]. Recent studies have indicated that PAs can be in large part effective to avoid the loss of forests within their boundaries [24, 75, 76]. However, human pressure in the rest of the landscapes continues increasing with the consequent increased isolation, decreased forest habitat in the PAs, and decreased capacity of the PAs to provide proper protection for biodiversity and maintenance of the ecosystem services [54, 64, 77].

The present study revealed significant changes in forest cover and the spatial patterns of the forests in the surrounding landscapes and, to a lesser extent, in the PAs. Based on this tendency and if the deforestation drivers continue operating, it is expected that the loss and fragmentation of the tropical Andean forest will continue in Ecuador during the next decades. Therefore, there is no doubt that deforestation will affect the biological diversity of the Chocó-Darien hotspot, which houses one of the greatest floristic diversity of the neotropical region and the world [78].

## Conclusion

The present study confirmed that the PAs are a shield against deforestation, especially in areas with high population density. However, these PAs cannot avoid the fragmentation of the surrounding forests. If this tendency continues in the future, it is expected that the biodiversity in the Chocó-Darién hotspot will be significantly affected.

It is worth mentioning the ability of remote sensors to provide quantitative information about the rate of deforestation and changes in spatial patterns. In addition, the present study emphasizes the need of interaction between the managers of the PAs and local communities, in order to lessen the economic dependence on the PAs and provide an alternative strategy for the generation of livelihoods and, in turn, involve human settlements in the management of the PAs.

Finally, it is clear that the efforts for conservation of the PAs and the surrounding landscapes produce positive results. However, greater efforts are still required to prevent that natural forests contained in the PAs become isolated and with little chance of connectivity.

## Supporting information

S1 File Statistical analysis of the metrics by class. U the Mann-Whitney was used to report significant differences in landscape metrics.
(R) Click here for additional data file.

S2 File Drivers inside Protected Areas 1990–2000. A generalized linear model was used to report drivers of deforestation within protected areas between 1990 and 2000.
(R) Click here for additional data file.

S3 File Drivers inside Protected Areas 2000–2008. A generalized linear model was used to report drivers of deforestation within protected areas between 2000 and 2008.
(R) Click here for additional data file.

S4 File Drivers inside Protected Areas 2008–2014. A generalized linear model was used to report drivers of deforestation within protected areas between 2008 and 2014.
(R) Click here for additional data file.

S5 File Drivers outside Protected Areas 1990–2000 (Surrounding area). A generalized linear model was used to report drivers of deforestation outside protected areas between 1990 and 2000.
(R) Click here for additional data file.

S6 File Drivers outside Protected Areas 2000–2008 (Surrounding area). A generalized linear model was used to report drivers of deforestation outside protected areas between 2000 and 2008.
(R) Click here for additional data file.

S7 File Drivers outside Protected Areas 2008–2014 (Surrounding area). A generalized linear model was used to report drivers of deforestation outside protected areas between 2008 and 2014.
(R) Click here for additional data file.

S8 File Statistical analysis of the metrics by class for two protected areas. U the Mann-Whitney was used to report significant differences in landscape metrics.
(R) Click here for additional data file.
